# The PACOVAR-trial: A phase I/II study of pazopanib (GW786034) and cyclophosphamide in patients with platinum-resistant recurrent, pre-treated ovarian cancer

**DOI:** 10.1186/1471-2407-11-453

**Published:** 2011-10-20

**Authors:** Michael Eichbaum, Christine Mayer, Regina Eickhoff, Esther Bischofs, Gerhard Gebauer, Tanja Fehm, Florian Lenz, Hans-Christian Fricke, Erich Solomayer, Nikos Fersis, Marcus Schmidt, Markus Wallwiener, Andreas Schneeweiss, Christof Sohn

**Affiliations:** 1 University of Heidelberg Medical School, Department of Gynecology and Obstetrics, Voss-Str. 9, 69115 Heidelberg, Germany; 2 Alcedis GmbH, Winchesterstraße 2, 35394 Gießen, Germany; 3 Klinik für Gynäkologie und gynäkologische Onkologie, Marienkrankenhaus Hamburg, Alfredstraße 9, 22087 Hamburg, Germany; 4 University of Tuebingen Medical School, Department of Gynecology and Obstetrics, Calwerstraße 7, 72076 Tuebingen, Germany; 5 Frauenklinik Krankenhaus Hetzelstift Neustadt, Stiftstraße 10, 67434 Neustadt an der Weinstraße, Germany; 6 Frauenklinik Klinikum Konstanz, Luisenstraße 7, 78464 Konstanz, Germany; 7 University of Homburg/Saar Medical School, Department of Gynecology and Obstetrics, 66424 Homburg/Saar, Germany; 8 Frauenklinik Klinikum Chemnitz gGmbH, Flemmingstraße 4, 09116 Chemnitz, Germany; 9 University of Mainz Medical School, Department of Gynecology and Obstetrics, Langenbeckstr. 1, 55131 Mainz, Germany; 10 National Center for Tumor Diseases, University Hospital, Im Neuenheimer Feld 460, 69120 Heidelberg, Germany

## Abstract

**Background:**

The prognosis of patients with recurrent, platinum-resistant epithelial ovarian cancer (EOC) is poor. There is no standard treatment available. Emerging evidence suggests a major role for antiangiogenic treatment modalities in EOC, in particular in combination with the metronomic application of low dose chemotherapy. The novel, investigational oral antiangiogenic agent pazopanib targeting vascular endothelial growth factor receptor (VEGFR), platelet-derived growth factor receptor (PDGFR) and c-kit is currently being studied in different tumour types and is already used as first line therapy in recurrent renal cell carcinoma. A combined therapy consisting of pazopanib and metronomic oral cyclophosphamide may offer a well-tolerable treatment option to patients with recurrent, pretreated EOC.

**Methods/design:**

This study is designed as a multicenter phase I/II trial evaluating the optimal dose for pazopanib (phase I) as well as activity and tolerability of a combination regimen consisting of pazopanib and metronomic cyclophosphamide in the palliative treatment of patients with recurrent, platinum-resistant, pre-treated ovarian cancer (phase II). The patient population includes patients with histologically or cytologically confirmed diagnosis of EOC, cancer of the fallopian tube or peritoneal cancer which is platinumresistant or -refractory. Patients must have measurable disease according to RECIST criteria and must have failed available standard chemotherapy. Primary objectives are determination of the optimal doses for pazopanib (phase I) and the overall response rate according to RECIST criteria (phase II). Secondary objectives are time to progression, overall survival, safety and tolerability. The treatment duration is until disease progression or intolerability of study drug regimen (with a maximum of 13 cycles up to 52 weeks per subject).

**Discussion:**

The current phase I/II trial shall clarify the potential of the multitargeting antiangiogenic tyrosinkinaseinhibitor GW 786034 (pazopanib) in combination with oral cyclophosphamide as salvage treatment in patients with recurrent, pretreated ovarian cancer.

**Trial registration:**

ClinicalTrials.gov: NCT01238770

## Background

About 8,200 women a year in Germany develop a malignant tumour of the ovary. The incidence of ovarian carcinoma has remained unchanged in the last few decades [1]. With more than 6,000 deaths, it is the fifth highest cause of cancer-related mortality in women [2]. Symptoms of the disease usually develop at a very late stage. For this reason about 70% of patients are already in an advanced stage of the tumour at the time of diagnosis (FIGO III or IV). Surgical tumour removal is the primary treatment. Whether and to what extent residual tumour formation is present postoperatively is the deciding factor in the subsequent prognosis for the patient. After surgery, chemotherapy involving paclitaxel plus carboplatin is generally indicated in the event of an initially advanced tumour stage [3-5]. Despite improved surgical procedures and a high primary response to chemotherapy, about 75% of patients with advanced ovarian carcinoma develop a tumour relapse and die from the disease [6].

Data from numerous preclinical and clinical trials support the assumption of VEGF/VEGFR and PDGFR as target molecules for the treatment of the ovarian cancer. Angiogenesis is a critical pathway in the development and progression of cancer. Therefore, identification and development of novel agents with limited toxicity that target mechanisms of tumor progression such as angiogenesis is of high priority.

Metronomic chemotherapy, defined as the frequent administration of low doses of cytotoxic chemotherapy at frequent intervals, suppresses tumor growth in experimental models, possibly by inhibiting angiogenesis by stimulating the release of thrombospondin [7-9]. These experimental findings are supported by a clinical trial where encouraging activity with minimal toxicity was observed in patients with breast cancer [10]. In 2007, Samaritini and co-workers reported about cyclophosphamide "metronomic" chemotherapy for palliative treatment of a young patient with advanced epithelial ovarian cancer. They demonstrated that the progression-free survival time on daily low dose oral cyclophosphamide treatment was 65 months without side effects [11]. Furthermore, in experimental models, the combined use of metronomic chemotherapy with antiangiogenic therapies demonstrates marked inhibition of tumor growth [12-15].

Recent data have shown that the combination of bevacizumab and metronomic oral cyclophosphamide, has encouraging activity in recurrent ovarian cancer [16-18]. Further studies of synergistical effects to cut off the blood supply by combination of known anti-angiogenesis agents with a lower dose of chemotherapy on a prolonged basis is warranted.

Pazopanib is an oral, angiogenesis inhibitor targeting vascular endothelial growth factor receptor (VEGFR), platelet-derived growth factor receptor (PDGFR) and c-kit. VEGF and PDGF are growth factors critical to the development and growth of blood vessels - a process known as angiogenesis. Angiogenesis plays a pivotal role in the growth and spread of several tumour types, with VEGF and PDGF overexpression linked to multiple cancers including cancers of the liver, lung, breast, kidney, bladder, ovaries, and colon [19-21]. By inhibiting VEGFR, PDGFR, and c-kit pazopanib may stop or slow the rate of tumour growth and development. Pazopanib is currently being studied in a number of different tumour types; clinical data are published in renal cell carcinoma (Phase III), breast cancer, ovarian cancer, STS, NSCLC, cervical cancer and clinical trials are currently underway in other solid tumours. It is being evaluated as a monotherapy, in combination with targeted therapies and in combination with cytotoxic chemotherapy. More than 2,000 patients have been treated to date in clinical trials. Pazopanib (under the tradename Votrient^®^) has been licensed by the FDA for the treatment of patients with advanced renal cell carcinoma (RCC) on the 19^th ^of Oct 2009 and is on the market in the US. In the EU, pazopanib has been given a positive opinion by the committee for proprietary medicinal products (CPMP) at the European Medicines Agency 19^th ^February 2010. Pazopanib (under the tradename Votrient^®^) has been licensed by EMA in EU on the 14^th ^of June 2010 for the treatment of patients with advanced renal cell carcinoma (RCC).

Pazopanib demonstrated manageable safety profile and clinical activity in a phase I study including ovarian cancer tumor types [22]. Clinical efficacy of pazopanib treatment has been seen in several Phase II/III studies, including in renal cell cancer [23], soft tissue sarcoma [24], breast [25], non-small cell lung cancer [26] and cervical cancer [27]. Study VEG104450 provided proof-of-concept data for pazopanib as monotherapy in ovarian cancer. VEG104450 is a Phase II study of pazopanib in subjects with ovarian, fallopian tube, or primary peritoneal cancers who had responded to first-line chemotherapy and who were at high risk of clinical recurrence (as evidenced by rising CA-125 levels). 36 subjects were enrolled, of which 21 (58%) have received one prior chemotherapy regimen. Final results obtained recently indicate the following: 11 out of 36 (31%) subjects experienced a CA-125 response to pazopanib with responses occurring shortly after start of pazopanib administration (median time to response 29 days) with a median duration of response of 113 days [28].

The current trial shall clarify the potential of the multitargeting antiangiogenic tyrosinkinaseinhibitor GW 786034 (pazopanib) in combination with oral cyclophosphamide as salvage treatment in patients with recurrent, pretreated ovarian cancer.

## Methods/design

### Study design

This study is designed as a multicenter phase I/II trial evaluating the optimal dose for pazopanib (phase I) as well as activity and tolerability of a combination regimen consisting of pazopanib and metronomic cyclophosphamide in the palliative treatment of patients with recurrent, platinum-resistant, pre-treated ovarian cancer (phase II).

### Study objectives

#### Primary Objectives

• Determination of the optimal dose for pazopanib (phase I)

• Determination of the overall response rate according to RECIST criteria/clinical benefit (stable disease or partial response or complete response) (phase II) 12 weeks after start of treatment

#### Secondary Objectives

• Time to progression (TTP) according to RECIST criteria

• Overall survival

• Evaluation of CA125 tumour response

• Safety and tolerability

• Assessment of quality of life over time as defined by EORTC-QLQ C 30 and Ovar 28 questionnaire

### Trial organization

The PACOVAR trial has been designed by the study initiators at the Department of Gynecology and Obstetrics at the University of Heidelberg Medical School. The trial is carried out as a multicenter trial under cooperation with the Department of Gynecology and Obstetrics of the University of Tuebingen Medical School, the Department of Gynecology and Gynecologic Oncology of Marienkrankenhaus Hamburg, the Department of Gynecology and Obstetrics of the University of Homburg/Saar, the Department of Gynecology and Obstetrics, Klinikum Hetzelstift Neustadt, the Department of Gynecology and Obstetrics, Klinikum Konstanz, the Department of Gynecology and Obstetrics of the University of Mainz Medical School and the Department of Gynecology and Obstetrics, Klinikum Chemnitz.

### Coordination

The overall coordination is performed by the Department of Gynecology and Obstetrics at the University of Heidelberg Medical School. This department is also responsible for the overall trial management, database management, quality assurance including monitoring and reporting.

### Investigators

The study investigators are experienced gynecolgic oncologists. Patients will be recruited and treated by the phycisians of the corresponding trial center.

### Ethics, informed consent and safety

The final protocol was approved by the ethics committee of the University of Heidelberg, Heidelberg, Germany (AFmu-241/2010). This study complies with the Helsinki Declaration in its recent German version, the Medical Association code of conduct, the principles of Good Clinical Practice (GCP) and the Federal Data Protection Act. The trial will also be carried out in keeping with local legal and regulatory requirements. The medical secrecy and the Federal Data Protection Act will be followed. The http://ClinicalTrials.gov Protocol ID is NCT01238770.

### Patient selection

Patients may be included in the study only if they meet all the following criteria:

1. Subjects must provide written informed consent prior to performance of study specific procedures or assessments, and must be willing to comply with treatment and follow up assessments and procedures

2. Female subjects > 18 years of age

3. Histologically or cytologically confirmed diagnosis of: epithelial ovarian cancer which is platinum resistant (relapse-free interval < 6 months of a platinum-containing primary or secondary platinum therapy) or platinum refractory (progressive disease during primary or secondary platinum therapy), cancer of the fallopian tube, peritoneal cancer. *Definition of relapse: *Demonstration of measurable or non-measurable tumour according to RECIST criteria by an imaging procedure (where applicable before relapse surgery) *or *increase in the tumour marker CA-125 to twice the upper laboratory value of normal for the hospital

4. Patients must have failed available standard chemotherapy regimen (except if medically contraindicated or refused by the patient)

5. Prior treatment with at least 2 chemotherapy regimens in advanced tumor setting

6. Performance status ECOG 0-2

7. Adequate contraception

8. Adequate organ function as defined

9. Measurable disease according to RECIST criteria

10. Able to swallow and retain oral medication

11. A life expectancy of at least 12 weeks

### Excluson criteria

Patients will be excluded from the study for any of the following reasons:

1. Diagnosis of any second malignancy within the last 5 years, with the exception of basal cell or squamous cell skin cancer or in situ carcinoma of the cervix uteri

2. History or clinical evidence of central nervous system (CNS) metastases or

leptomeningeal carcinomatosis, except for individuals who have previously-treated CNS metastases, are asymptomatic, and have had no requirement for steroids or anti-seizure medication for 6 months prior to first dose of study drug. Screening with CNS imaging studies (computed tomography [CT] or magnetic resonance imaging [MRI]) is required only if clinically indicated or if the subject has a history of CNS metastases.

3. Clinically significant gastrointestinal abnormalities which might interfere with oral dosing

• Active peptic ulcer disease

• Known intraluminal metastatic lesion/s with suspected bleeding

• Inflammatory bowel disease

• Ulcerative colitis, or other gastrointestinal conditions with increased risk of perforation

• History of abdominal fistula, gastrointestinal perforation, or intraabdominal abscess within 28 days prior to beginning study treatment

• Malabsorption syndrome

• Major resection of the stomach or small bowel

4. Any unstable or serious concurrent condition (e.g., active infection requiring systemic therapy)

5. Prolongation of corrected QT interval (QTc) > 480 msecs

6. History of any one or more of the following cardiovascular conditions within the past 6 months:

• Cardiac angioplasty or stenting

• Myocardial infarction

• Unstable angina

• Symptomatic peripheral vascular disease

• Coronary artery by-pass graft surgery

• Class II, III or IV congestive heart failure as defined by the New York Heart Association (NYHA)

• History of cerebrovascular accident, pulmonary embolism or untreated deep venous thrombosis (DVT) within the past 6 months. Note: Subjects with recent DVT who have been treated with therapeutic anticoagulant agents (excluding therapeutic warfarin) for at least 6 weeks are eligible

7. Macroscopic hematuria

8. Hemoptysis that is clinically relevant within 4 weeks of first dose of study drug

9. Evidence of active bleeding or bleeding diathesis

10. Known endobronchial lesions or involvement of large pulmonary vessels by tumor

11. Prior major surgery or trauma within 14 days prior to first dose of study drug and/or presence of any non-healing wound, fracture, or ulcer

12. Chemotherapy or radiation therapy within 2 weeks prior to the first dose of study drug.

13. Biological therapy, hormonal therapy or treatment with an investigational agent within 28 days or 5 half-lives, whichever is longer prior to the first dose of study drug.

14. Prior antiangiogenic therapy.

15. Is unable or unwilling to discontinue predefined prohibited medications listed in the protocol for 14 days or five half-lives of a drug (whichever is longer) prior to Visit 1 and for the duration of the study

16. Any ongoing toxicity from prior anti-cancer therapy that is > Grade 1 and/or that is progressing in severity

17. Known immediate or delayed hypersensitivity reaction or idiosyncrasy to drugs chemically related to pazopanib

18. Psychological, familial, sociological, or geographical conditions that do not permit compliance with the protocol.

19. Pregnancy (for women of childbearing potential absence to be confirmed by ß-hCG-test) or lactation period or missing contraception of women of childbearing potential (Pearl-Index < 1, e.g. hormonal contraception including the combined oral contraceptive pill, the transdermal patch, and the contraceptive vaginal ring, intrauterine devices or sterilization) during treatment and for at least 6 months thereafter

20. More than 3 different chemotherapy regimens in advanced tumor setting

21. Uncontrolled hypertension

22. History of ischemic event (stroke, myocardial infarction, unstable angina, TIA, symptomatic peripheral vascular disease)

23. History or clinical evidence of thrombo-embolic event

24. History of haemoptysis, cerebral, or clinically significant gastrointestinal haemorrhage in the past 6 months

25. Active bleeding

26. Signs/Suspicion of intestinal obstruction

### Statistical calculations for trial sample size

#### Phase I

The sample size of the phase I part is based on the need to establish the MTD of pazopanib in combination with cyclophosphamide. Dependent on the dose escalation, as many as 18 patients may be treated.

#### Phase II

The primary endpoint of phase II is the quantification of the responder-fraction (CR/PR) according to RECIST criteria of patients with platinum-resistant recurrent, pre-treated ovarian cancer who receive pazopanib and cyclophosphamide after 12 weeks of therapy. For each patient the objective response is determined. Complete and partial remission are considered as showing a response, otherwise there is no response.

The sample size was determined by using Simon's two stage optimal design [29]. The optimal design is that design which attempts to ensure that as few patients as possible receive what appears to be an ineffective drug at the end of Stage I for the given constraints. In order to calculate the sample size the following hypothesis was considered

H0: p≤0.10 versus H1: p≥0.30,

meaning that a response rate of at most 10% of the new therapy would have no clinical relevance whereas a response rate of at least 30% would lead to further investigation of that therapy. Furthermore the significance level will be set to 5% and the power to 90% which leads to the following sample sizes and stop criteria.

Table 1 shows the main statistical parameters of the trial calculation.

**Table 1:** Main statistical parameters of the trial calculation

Parameter	
p_0_	10%

p_1_	30%

α	5%

β	10%

Number of patients	
Stage I	18
Stage II	17

Rejection of therapy	
Stage I	0 - 2 responder among 18 patients
Stage II	0 - 6 responder among 35 patients

The above mentioned numbers refer to the sample size of the Full analysis set. Assuming 10% attrition and non-evaluability, this enrolment should yield 39 patients for analysis. Patients who discontinue participation in the clinical study will not be replaced as attrition and non-evaluability are already considered in the calculation of sample size.

### Study Medication

#### Cyclophophamide

Cyclophosphamide is a nitrogen mustard alkylating agent, from the oxazophorines group. Cyclophosphamide mustard forms DNA crosslinks between (interstrand crosslinkages) and within (intrastrand crosslinkages) DNA strands at guanine N-7 positions. This leads to cell death.

Exhibiting cytotoxic effects only after activation in the liver, cyclophosphomid is called a prodrug. Cyclophosphamide is converted by enzymes of the Cytochrome P450 to active metabolites. The main active metabolite is 4-hydroxycyclophosphamide, which exists in equilibrium with its tautomer, aldophosphamide. Most of the aldophosphamide is oxidised by the enzyme aldehyde dehydrogenase (ALDH) to make carboxyphosphamide. Therefore, cells with relatively large concentrations of aldehyde dehydrogenase (ALDH) (e.g. earlyhaematopoetic stem cells and megakaryocytes as well as stemcells of the mucous membranes) are less sensitive to the toxic effects of cyclophoshamid than cells with lower levels of aldehyde dehydrogenase. This difference in metabolism is the reason for the relatively low toxicity of bone marrow (as anaemia, thrombocytopenia and leukopenia) or mucous membranes.

#### Pazopanib

Pazopanib is an, oral, angiogenesis inhibitor targeting vascular endothelial growth factor receptor (VEGFR), platelet-derived growth factor receptor (PDGFR) and c-kit. VEGF and PDGF are growth factors critical to the development and growth of blood vessels - a process known as angiogenesis. Angiogenesis plays a pivotal role in the growth and spread of several tumour types, with VEGF and PDGF overexpression linked to multiple cancers including cancers of the liver, lung, breast, kidney, bladder, ovaries, and colon. By inhibiting VEGFR, PDGFR, and c-kit pazopanib may stop or slow the rate of tumour growth and development. Pazopanib is currently being studied in a number of different tumour types; clinical trials are currently underway in renal cell carcinoma (Phase III), breast cancer, ovarian cancer, STS, NSCLC, cervical cancer and other solid tumours. It is being evaluated as a monotherapy, in combination with targeted therapies and in combination with cytotoxic chemotherapy. More than 2000 patients have been treated to date in clinical trials. Pazopanib (under the tradename Votrient^®^) has been licensed by the FDA for the treatment of patients with advanced renal cell carcinoma (RCC) on the 19^th ^of Oct 2009 and is on the market in the US. In the EU, pazopanib has been given a positive opion by the committee for proprietary medicinal products (CPMP) at the European Medicines Agency 19^th ^February 2010. Pazopanib (under the tradename Votrient^®^) has been licensed by EMA in EU on the 14^th ^of June 2010 for the treatment of patients with advanced renal cell carcinoma (RCC).

### Adverse events

#### Cyclophosphamide

The most common dose-limiting toxicity of cyclophosphamide is myelosuppression.

Under therapy with Endoxan^® ^(trade name of cyclophosphamide) the following, mostly reversible side effects may occur dosisdependently. As a consequence, in a metronomic low-dose application all stated adverse events are seldom:

##### Blood and bone marrow

Myelosuppression with leukocytopenia, thrombocytopenia and anaemia in different grades of severity. Common: leukocytopenia with or without fever and the risk of secondary (to some extend life threatening) infections, thrombocytopenia, enhanced risk of bleeding

##### Gastro-intestinal (stomach and bowel) disorders

Common: Nausea, vomiting, anorexia, diarrhea, obstipation and stomatitis.

Uncommon: Haemorraghic colitis and ulceration of the oral mucosa.

##### Renal and urinary disorders

Common: Haemorraghic cystitis, micro-haematuria, macrohaematuria.

##### Hepato-biliary disorders

Uncommon: Increase in SGOT, SGPT, gamma-GT, alcalic phosphatase, bilirubin.

##### Cardiac, respiratory, thoracic and mediastinal disorders

Following high doses of cyclophosphamide (120-240 mg/kg body weight: Secondary cytostatics-induced cardiomyopathy (arrhythmia, ECG alterations, LVEF alterations (e.g. myocardial infarction). Following conventional doses of cyclophosphamid: Uncommon: Pneumonitis, interstitial pneumonia, chronic interstitial lung firbrosis.

##### Secondary malignancies

As generally described for cytostatic therapy, for the application of cyclophosphamide risc of secondary malignancy or prestages are described as long-term complications due to therapy. Enhanced risk exists e.g. for developement of carcinoma of urinary tract as well as for myelodysplastic alterations up to acute leukaemia.

##### Metabolic disorders

In particular in patients with non-hodgkin-lymphoma or leukaemia, increased levels of uric acid may occur due to a rapid-tumor-lysis syndrome.

##### Other side effects

Common: Alopecia (reversible).

Also alterations of pigment of palm of hand, finger nails, sole of foot as well as skin inflammation and mucositis are reported.

#### Pazopanib

In clinical trials, pazopanib has been well tolerated. Its most common side effects in clinical trials to date include diarrhea, hypertension, fatigue, nausea, anorexia, vomiting, hair depigmentation, rash and abdominal cramps. More specifically, in the phase I clinical trial in patients with solid tumors, the most common adverse events (all grades) were hypertension (33%) diarrhea (33%), hair depigmentation (32%), nausea (32%), anorexia (25%), fatigue (24%) andvomiting (17%), [22]; hypertension and hair depigmentation were indicative of VEGFR and c-kit modulation.

In the clinical trial in patients with gynecological cancers, fatigue, hypertension, diarrhea, ALT/AST elevation,, vomiting, abdominal pain and nausea were the most common side effectsGrade 3 adverse events were fatigue (11%), GGT elevation (11%), diarrhea (8%), ALT elevations (8%), Hypertension (3%), AST elevation (3%), Abdominal pain (3%), Constipation (3%), Headache (3%) and Ascites (3%). Only one grade 4 adverse event (peripheral edema) was reported; [28].

### Investigation schedule

#### Therapy indication

This study is a prospective open-label, non-randomized multicenter phase I/II trial in order to determine overall response rate of patients with platinum-resistant or refractory recurrent, pretreated epithelial ovarian cancer.

#### Pre-therapeutic examinations

Patients eligible for the study will initially be screened for their suitability to take part in the study. Patients not included in the study should be recorded in a screening log, with a statement of the reasons for their non-participation. Informed consent must be obtained prior to performance of any study-specific procedure. If not noted otherwise, the baseline examinations should be performed within 4 weeks before start of treatment:

• Previous history, physical examination including weight control and vital signs, ECG (ECG no more than 21 days old on inclusion in the study), gynaecological examination

• Assessment of general well-being according to ECOG within 7 days prior to start of study drug

• Documentation of measurable and non-measurable tumour lesions. For radiological disease assessments, CTs and MRIs are acceptable and are used interchangeably below; however, for each individual subject the same one method must be used throughout the study.

• CA-125 tumour marker (determined not more than 7 days before start of treatment). Two pretreatment values at least twice the upper limit of normal are required for an evaluation of the response by means of CA-125.

• Blood count, blood chemistry obligatory (within 7 days before inclusion in the study and within 4 weeks before start of treatment): Haematocrit, haemoglobin, white blood cell (WBC) count, red blood cell (RBC) count, lymphocyte count, neutrophil count, platelet count, aPTT and PT-INR, SGOT, SGPT, alkaline phosphatase, bilirubin (total), urea (blood urea nitrogen), creatinine, creatinine clearance (CrCl) calculated using Cockcroft and Gault formula, calcium, potassium, sodium, magnesium, glucose, lactate dehydrogenase (LDH), albumin, amylase, TSH, free T4, urine dipstick for protein

• Women of childbearing potential must have a negative pregnancy test (HCG in urine/serum) not more than 7 days old on start of treatment.

Quality of life (EORTC-QLQ C 30 and Ovar 28)

### Treatment plan

#### Phase I

   Cyclophosphamide   Pazopanib

Dose level I   50 mg/day   400 mg/day

Dose level II   50 mg/day   600 mg/day

Dose level III   50 mg/day   800 mg/day

Within the phase I part of the trial, doses of cyclophosphamide and pazopanib are assigned at registration according to the dose escalation scheme following Goodman et al. [30].

Before embarking on the phase II part of the study, phase I safety data will be reviewed. If there is a dose-limiting toxicity at dosage level I, then the phase II part of the study will not be initiated.

#### Treatment Regimen Phase II

Cyclophosphamide 50 mg daily orally.

Pazopanib 400, 600 or 800 mg daily orally.

The dose for the phase II part of the trial will be based on the MTD of phase I.

#### Duration of treatment

A treatment cycle consists of 4 weeks. Patients will receive up to 13 cycles (52 weeks) of cyclophosphamid with pazopbanib. Patients continue on treatment until until disease progression or intolerability of study drug regimen or as long as no other therapy is indicated.

### Tumour assessment and response

#### Tumour Response According to RECIST Criteria

Target lesions will be measured in a dimension using the longest diameter. The tumour response will be investigated every 12 weeks during treatment phase and every three months during Follow-Up or on signs of tumour progression. The same investigational method must be used throughout. Any partial or complete remission must be confirmed after at least 4 weeks. Complete response (CR): Disappearance of all lesions and appearance of no new lesions. Partial response (PR): At least a 30% decrease in the sum of the longest diameter of target lesions. No new lesions may occur or individual lesions progress. Progressive disease (PD): At least a 20% increase in the total of the longest diameters of target lesions (as reference to the smallest sum recorded since the treatment started) or the appearance of new lesions. If the marker lesion disappears and the progression of a lesion or the appearance of new lesions is observed elsewhere at the same time, this is also documented as a progressive disease.

Stable disease (SD): Neither a partial nor a complete response in the absence of progression. Confirmation of response is required in the case of CR and PR at a minimum interval of not less than 4 weeks. In the case of SD, measurements must have met the SD criteria at least once after study entry at a minimum interval of not less than 6-8 weeks.

#### CA-125 Tumour Response

Based on the CA-125 response criteria of the GCIG [31], modified according to Levy et al. [32], the following definitions are employed:

Complete response (CR): The CA-125 value of two determinations falls to within the normal range for at least 4 weeks. Partial response (PR): A decrease in the CA-125 value by ≥ 50% in 2 determinations for at least 4 weeks. Progressive disease (PD): An increase in CA-125 to values ≥ 125% of baseline value 2. At the same time, tumour lyses must be excluded clinically. Stable disease (SD): Patients to whom none of the previous criteria apply for at least 8 weeks.

Response unevaluable: Defined as **no **new evaluation of the tumour by the determination of CA-125 after the beginning of the study therapy *for reasons not associated with the symptoms or signs of the disease*.

After a complete remission is obtained, a (secondary) progression is defined as an increase in CA-125 to ≥ 125% of the upper limit of normal. After a partial remission or stable disease, a progression is present if the CA-125 increases to more than 125% of the nadir. The flow chart for the determination of CA-125 and for assessing the response

### Assessment of safety

#### Adverse Events

The investigator is responsible for documenting all adverse events that occur during the study. Adverse events are all harmful, pathological or unintentional changes in anatomical, physiological or metabolic functions observable in the form of physical signs, symptoms and/or changes in laboratory values that occur during any part of the clinical trial, irrespective of whether there is any relationship to a medication. This also includes a deterioration in preexisting diseases or events, diseases that occur in the intervening period, changes of medication or a significant deterioration in the disease studied. Expected daily variations in the disease under study that do not represent a clinically significant deterioration should not be considered an adverse event.

#### Causal Assessment

The investigator should make every effort to elucidate any adverse event and where necessary to assess its relationship to the study medication. The causal relationship should be assessed on the basis of the following categories: no relationship, relationship unlikely, relationship suspected (justified suspicion) and relationship probable. The degree of certainty of the relationship between adverse event and drug is determined by how well the event can be explained by the following ftors: (1) known pharmacological properties, (2) comparable reactions observed previously with the drug or another member of its class, (3) an event with comparable substances commonly described in the literature as drug-related, (4) a chronological relationship between the event and drug intake, disappearance on withdrawal or recurrence on re-institution of the drug.

#### Follow-up

Follow-up investigations will be done every 3 months over 24 months with the aim of determining the time of tumour progression.

The following examinations will be performed every 3 months after end of treatment

• Survival status

• Medical history, physical examination

• Assessment of ECOG performance

• Determination of tumour progression

• CA-125 tumour marker

• Further anticancer treatment

• Quality of life (EORTC-QLQ C 30 and Ovar 28)

The follow-up information can be collected by phone.

#### Monitoring

Study monitoring is undertaken by monitors appointed by ALCEDIS GMBH, Giessen, Germany. The responsible monitor will be allowed, on request, to inspect the various records of the trial (Case Report Forms and other pertinent data). Due to the electronic documentation system checks for range and plausibility are performed during data entry. The monitor gets an access to read the data only. In line with ICH GCP guidelines, monitoring will include verification of data entered in the eCRF against original patient records. This verification will be performed by direct access to the original subject records, and the Sponsor guarantees that patient confidentiality will be respected at all times. Participation in this study will be taken as agreement to permit direct source data verification.

## Discussion

Epithelial ovarian cancer remains the most lethal gynecologic malignancy [1-4,6]. In recurrent, advanced disease, treatment options are limited after failure of platinum compounds and new investigational strategies are highly needed [1-6].

There is increasing evidence that antiangiogenic targeted therapies show promising efficacy in the palliative systemic treatment of the disease. In particular bevacizumab, combined with metronomically administered cyclophosphamide was shown to be beneficial [16-18]. It is the aim of this trial to elucidate the therapeutic potential of the new antiangiogenically active multityrosinekinase inhibitor pazopanib combined with metronomically orally taken low dose cyclophosphamide in patients with advanced EOC. Once the best tolerable dosage has been found within the first phase I-part of the trial, a subsequent phase II-investigation shall clarify the activity and tolerability of the schedule in a larger number of patients.

If a combination of pazopanib and metronomic low dose cyclophosphamide can be shown to be active and feasible in intensively pretreated patients with advanced EOC this would mark a major clinical benefit as a new, additive and presumably well mangeable oral treatment option could be add to the existing therapy concepts and give time and quality of life to our patients.

## Abbreviations

ADR: Adverse Drug Reactions; ALDH: Aldehyde dehydrogenases; ALT: Alaninaminotransferase; Ca 125: Cancer Antigen 125; CNS: Central nervous system; CPMP: Committee for proprietary medicinal products; CR: Complete Remission; CT: Computer tomography; DNA: Deoxyribonucleic acid; DVT: deep vein thrombosis; ECG: electrocardiography; eCRF: electronic case report form; ECOG: Eastern cooperative oncology group; EMA: European Medicines Agency; EOC: Epithelial Ovarian Cancer; EORTC-QLQ 30 European Organization for Resaerch and treatment of Cancer-Qualitiy of life questionnaire; FDA: Food and Drug Administration; GCIG: Gynecologic Cancer Intergroup; GCP: Good Clinical Practice; hCG: human chorionogonadotropin; LVEF: left ventricular ejection fraction; MRI: Magnet resonance imaging; MTD: Maximum Tolerated Dosage; NC: No Change; NSCLC: Non small cellular lung cancer; NYHA: New York Heart Association; ORR: Overall Response rate; PD: Progressive Disease; PDGF: Platelet derived growth factor; PDGFR: Platelet derives growth factor receptor; PR: Partial Remission; RBC: Red blood cell; RCC: Renal cell carcinoma; RECIST: Response Evaluation Criteria In Solid Tumors; SCS: Safety critical systems; SD: Stable Disease; SGOT: Serum Glutamat-Oxalacetat transaminase; SGPT: Serum Glutamat-Pyruvat Transaminase; STS: Soft tissue sarcoma; TTP: Time-to-progression; VEGF: Vascular Endothelial Growth Factor; VEGFR: Vascular Endothelial Growth Factor Receptor; WBC: White blood cell.

## Competing interests

The authors declare that they have no competing interests.

## Authors' contributions

ME, CM, EB, MW, AS and CS planned, organized and conduct the trial. ME, CM, EB, MW, AS, CS, GG, MS, NF, ES, HCF recruit patients for the study. Medical care and follow-up is provided by all authors. All authors have read and approved the final version of the protocol.

## Pre-publication history

The pre-publication history for this paper can be accessed here:

http://www.biomedcentral.com/1471-2407/11/453/prepub

## References

[B1] GEKID-Atlas: Inzidenz und Mortalität von Krebserkrankungen in den Bundesländern http://www.ekr.med.uni-erlangen.de/GEKID/Atlas/CurrentVersion/Inzidenz/atlas.html

[B2] GEKID-Atlas: Inzidenz und Mortalität von Krebserkrankungen in den Bundesländern http://www.ekr.med.uni-erlangen.de/GEKID/Atlas/CurrentVersion/Mortalitaet/atlas.html

[B3] McGuire WP Brady MF Ozols RF The Gynecologic Oncology Group experience in ovarian cancer Ann Oncol 1999 10 29 34 10219450 10.1023/a:1008303300675

[B4] McGuire WP Primary therapy of epithelial ovarian cancer Amer Soc Clin Oncol Educational Book 2001 Spring 477 480

[B5] Parmar MK Ledermann JA Colombo N du Bois A Delaloye JF Kristensen GB Wheeler S Swart AM Qian W Torri V Floriani I Jayson G Lamont A Trope C Paclitaxel plus platinum-based chemotherapy versus conventional platinum-based chemotherapy in women with relapsed ovarian cancer: the ICON4/AGO-OVAR-2.2 trial Lancet 2003 361 9375 2099 106 12826431 10.1016/s0140-6736(03)13718-x

[B6] Colombo N Van Gorp T Parma G Amant F Gatta G Sessa C Vergote I Ovarian cancer Crit Rev Oncol Hematol 2006 60 2 159 79 10.1016/j.critrevonc.2006.03.004 17018256

[B7] Gately S Kerbel R Antiangiogenic scheduling of lower dose cancer chemotherapy Cancer 2001 7 427 436 11693902

[B8] Man S Bocci G Francia G Antitumor effects in mice of low dose (metronomic) cyclyophoshphamide administered continuously through the drinking water Cancer Res 2002 62 2731 2735 12019144

[B9] Bocci G Francia G Man S Thrombospondin 1, a mediator of the antiangiogenic effects of low-dose metronomic chemotherapy Proc Natl Acad Sci USA 2003 100 12917 12922, 10.1073/pnas.2135406100 14561896 PMC240719

[B10] Colleoni M Rocca A Sandri T Low dose oral methotrexate and cyclophosphamide in metastatic breast cancer: Antitumor activity and correlation with vascular endothelial growth factor levels Ann Oncol 2002 13 73 80 10.1093/annonc/mdf013 11863115

[B11] Samaritini R Corrado G Vizza E Sbiroli C Cyclophosphamide "metronomic" chemotherapy for palliative treatment of a young patient with advanced epithelial ovarian cancer BMC Cancer 2001 7 65 10.1186/1471-2407-7-65 PMC1863429 17433113

[B12] Bello L Carrabba G Giussani C Low dose chemotherapy combined with an antiangiogenic drug reduces human glioma growth in vivo Cancer Res 2001 61 501 506 11606386

[B13] Klement G Baruchel S Rak J Continuous low dose therapy with vinblastine and VEGF receptor-2 antibody induces sustained tumor regression without overt toxicity J Clin Invest 2000 105 R15 24 10.1172/JCI8829 10772661 PMC517491

[B14] Klement G Huang P Mayer B Differences in therapeutic indexes of combination metronomic chemotherapy and an anti-vegfr-2 antibody in multidrug resistant human breastcancer xenografts Clin Cancer Res 2002 8 221 232 11801563

[B15] Takahashi N Haba A Matsuno F Antiangiogenic therapy of established tumors in human skin/severe combined immunodeficiency mouse chimeras by antiendoglin (CD105) monoclonal antibodies and synergy between antiendoglin antibody and cyclophosphamide Cancer Res 2001 61 846 854 11691802

[B16] Chura JC Van Iseghem K Downs LS Jr Bevacizumab plus cyclophosphamide in heavily pretreated patients with recurrent ovarian cancer Gynecol Oncol 2007 107 326 30 10.1016/j.ygyno.2007.07.017 17706754

[B17] Garcia AA Hirte H Fleming G Phase II clinical trial of bevacizumab and low-dose metronomic oral cyclophosphamide in recurrent ovarian cancer: a trial of the California, Chicago, and Princess Margaret Hospital phase II consortia J Clin Oncol 2008 26 76 82 10.1200/JCO.2007.12.1939 18165643

[B18] Jurado García JM Sánchez A Pajares B Pérez E Alonso L Alba E Combined oral cyclophosphamide and bevacizumab in heavily pre-treated ovarian cancer Clin Transl Oncol 2008 10 583 6 10.1007/s12094-008-0254-7 18796376

[B19] Folkman J Angiogenesis in cancer, vascular, rheumatoid and other disease Nat Med 1995 1 1 27 31 10.1038/nm0195-27 7584949

[B20] Spannuth WA Sood AK Coleman RL Angiogenesis as a strategic target for ovarian cancer therapy Nat Clin Pract Oncol 2008 5 4 194 204 18268546 10.1038/ncponc1051

[B21] Folkman J What is the evidence that tumors are angiogenesis dependent? J Natl Cancer Inst 1990 82 4 6 10.1093/jnci/82.1.4 1688381

[B22] Hurwitz HI Dowlati A Saini S Savage S Suttle AB Gibson DM Hodge JP Merkle EM Pandite L Phase I Trial of Pazopanib in Patients with Advanced Cancer Clin Cancer Res 2009 15 4220 4227 10.1158/1078-0432.CCR-08-2740 19509175

[B23] Sternberg CN Davis ID Mardiak J Szczylik C Lee E Wagstaff J Barrios CH Salman P Gladkov OA Kavina A Zarba JJ Chen M McCann L Pandite L Roychowdhury DF Hawkins RE Pazopanib in Locally Advanced or Metastatic Renal Cell: Carcinoma: Results of a Randomized Phase III Trial J Clin Oncol 2010 28 1061 1068 10.1200/JCO.2009.23.9764 20100962

[B24] Sleijfer S Ray-Coquard I Papai Z Le Cesne A Scurr M Schöffski P Collin F Pandite L Marreaud S De Brauwer A van Glabbeke M Verweij J Blay JY Pazopanib, a multikinase angiogenesis inhibitor, in patients with relapsed or refractory advanced soft tissue sarcoma: a phase II study from the European organisation for research and treatment of cancer-soft tissue and bone sarcoma group (EORTC study 62043) J Clin Oncol 2009 27 19 3126 32 10.1200/JCO.2008.21.3223 19451427

[B25] Slamon D Gomez H Amit O Richie M Pandite L Roychowdhury D Goodman V Updated Results From a Randomized Study in Patients With First-line ErbB2-positive Advanced or Metastatic Breast Cancer ESMO Congress 2008 1233 [abstract]

[B26] Altorki N Lane ME Bauer T Lee PC Guarino MJ Pass H Felip E Peylan-Ramu N Gurpide A Grannis FW Mitchell JD Tachdjian S Swann RS Huff A Roychowdhury DF Reeves A Ottesen LH Yankelevitz DF Phase II proof-of-concept study of pazopanib monotherapy in treatment-naive patients with stage I/II resectable non-small-cell lung cancer J Clin Oncol 2010 28 19 3131 7 10.1200/JCO.2009.23.9749 20516450

[B27] Monk BJ Mas Lopez L Zarba JJ Oaknin A Tarpin C Termrungruanglert W Alber JA Ding J Stutts MW Pandite LN Phase II, open-label study of pazopanib or lapatinib monotherapy compared with pazopanib plus lapatinib combination therapy in patients with advanced and recurrent cervical cancer J Clin Oncol 2010 28 22 3562 9 10.1200/JCO.2009.26.9571 20606083

[B28] Friedlander M Hancock KC Rischin D Messing MJ Stringer CA Matthys GM Ma B Hodge JP Lager JJ A Phase II, open-label study evaluating pazopanib in patients with recurrent ovarian cancer Gynecol Oncol 2010 119 32 37 10.1016/j.ygyno.2010.05.033 20584542

[B29] Simon R Optimal two-stage designs for phase II clinical trials Contr Clin Trials 1989 10 1 10 10.1016/0197-2456(89)90015-9 2702835

[B30] Goodman SN Zahurak ML Piantadosi S Some practical improvements in the continual reassessment method for phase I studies Stat Med 1995 14 1149 61 10.1002/sim.4780141102 7667557

[B31] Rustin GJS Use of CA-125 to assess response to new agents in ovarian cancer trials J Clin Oncol 2003 21 187 203 10.1200/JCO.2003.01.223 12743133

[B32] Levy T Inbar M Menczer J Grisaru D Glezerman M Safra T Phase II study of weekly topotecan in patients with recurrent or persistent epithelial ovarian cancer Gynecol Oncol 2004 95 686 90 10.1016/j.ygyno.2004.09.005 15581982

